# Risk prediction model for radiation pneumonitis in breast cancer radiotherapy based on dose–volume parameters combined with the neutrophil-to-lymphocyte ratio

**DOI:** 10.3389/fonc.2026.1740592

**Published:** 2026-03-10

**Authors:** Jianliang Zhou, Xiya Liu, Pengrong Lou, Jiming Yang, Qingtao Xu, Xuhao Dai, Wenting Lan, Jiangping Ren

**Affiliations:** 1 Radiotherapy and Chemotherapy Department, The First Affiliated Hospital of Ningbo University, Ningbo, Zhejiang, China; 2 Ningbo University Health Science Center, Ningbo, Zhejiang, China; 3 Radiology Department, The First Affiliated Hospital of Ningbo University, Ningbo, Zhejiang, China

**Keywords:** breast cancer, neutrophil lymphocyte ratio (NLR), predictive models, radiation pneumonitis, risk factors

## Abstract

**Purpose:**

To develop and validate a risk prediction model for radiation pneumonitis (RP) and radiation-induced pulmonary fibrosis (RIPF) following breast cancer radiotherapy by integrating the V40 dose–volume parameter with the neutrophil-to-lymphocyte ratio (NLR), providing guidance for individualized treatment strategies.

**Methods:**

This retrospective cohort study analyzed clinical data from 164 patients with breast cancer who underwent postoperative radiotherapy between May 2018 and August 2020. Clinical–pathological characteristics, radiotherapy dosimetric parameters and NLR values were collected at three time points: pre-surgery, 1 week before radiotherapy and 1 month after radiotherapy. Radiation pneumonitis (0–6 months) and RIPF (≥6 months) were assessed according to the Common Terminology Criteria for Adverse Events (version 5.0). Receiver operating characteristic (ROC) curves were used to determine the optimal predictive indicators. Variable selection was performed using least absolute shrinkage and selection operator regression followed by multivariate logistic regression to construct the prediction model. Internal validation was conducted using 1,000 bootstrap resampling iterations.

**Results:**

Of the 164 patients, 107 (65.2%) developed varying degrees of RP (grade 1: n = 103, 62.8%; grade 2: n = 4, 2.4%), and 118 (72.0%) developed RIPF (all grade 1). The ROC analysis revealed that ipsilateral lung V40 had superior predictive performance for RIPF (area under the curve [AUC] = 0.728, 95% confidence interval [CI]: 0.651–0.805, cutoff value: 10.45%). The pre-radiotherapy NLR showed significant predictive value for RP (AUC = 0.685, 95% CI: 0.605–0.765, cutoff value: 2.82). Multivariate analysis identified independent risk factors for RP: V40 ≥ 10.45% (odds ratio [OR] = 3.24, 95% CI: 1.78–5.89, P < 0.001), pre-radiotherapy NLR ≥ 2.82 (OR = 2.56, 95% CI: 1.42–4.61, P = 0.002) and regional nodal irradiation (OR = 2.13, 95% CI: 1.18–3.84, P = 0.012). The combined prediction model achieved an AUC of 0.816 (95% CI: 0.748–0.884), significantly outperforming single indicators (ΔAUC = 0.088–0.131, P < 0.05). Bootstrap internal validation demonstrated robust model stability (C-index = 0.803).

**Conclusions:**

The integrated prediction model combining V40 and the NLR effectively identifies patients a high risk of RP following breast cancer radiotherapy, facilitating personalized treatment planning and early intervention strategies.

## Introduction

Breast cancer represents the most prevalent malignancy among women worldwide, with approximately 2.26 million new cases diagnosed annually, surpassing lung cancer as the most commonly diagnosed cancer globally (1). Radiotherapy constitutes an integral component of comprehensive breast cancer management, demonstrating efficacy in reducing locoregional recurrence rates by 15%–20% and improving overall survival by 5%–8% in appropriately selected patients (2). The Early Breast Cancer Trialists’ Collaborative Group meta-analysis confirmed that radiotherapy after breast-conserving surgery (BCS) reduces the 10-year recurrence risk and 15-year breast cancer mortality, establishing its role as a cornerstone of multimodal treatment (3). However, radiation pneumonitis (RP) has emerged as a substantial dose-limiting toxicity following thoracic radiotherapy, with reported incidence rates ranging from 5% to 30% depending on the treatment technique, dose–volume parameters and patient-specific factors (4).

The clinical spectrum of RP varies considerably, from asymptomatic radiographic changes detected incidentally on surveillance imaging to life-threatening respiratory compromise requiring intensive medical intervention (5). The pathophysiology involves an initial inflammatory phase characterized by endothelial damage and cytokine release, followed by a fibrotic phase with collagen deposition and the architectural distortion of lung parenchyma (6). Early identification of patients at elevated risk of developing RP remains crucial for optimizing radiotherapy planning, implementing preventive measures and ensuring timely therapeutic interventions that can mitigate long-term pulmonary sequelae (7).

Traditional risk stratification approaches have predominantly relied on dosimetric parameters derived from dose–volume histograms, including the mean lung dose (MLD) and the percentage of lung volume receiving specific radiation doses (V5, V10, V20, V30) (8). These parameters reflect the physical distribution of the radiation dose within lung tissue and have been validated across multiple studies as predictors of RP risk (9). However, these conventional metrics demonstrate limited predictive accuracy when applied in isolation, with area under the curve (AUC) values typically ranging from 0.60 to 0.70, necessitating the integration of additional biomarkers to enhance risk assessment precision (10).

Recent investigations have highlighted the pivotal role of systemic inflammation in radiation-induced tissue injury and subsequent fibrotic transformation (11). The inflammatory cascade triggered by radiation exposure involves complex interactions between immune cells, cytokines and growth factors that collectively determine the tissue response and repair capacity (12). Among various inflammatory biomarkers, the neutrophil-to-lymphocyte ratio (NLR) has emerged as a readily accessible and reproducible marker of systemic inflammatory response (13). The NLR reflects the dynamic balance between neutrophil-mediated pro-inflammatory responses and lymphocyte-associated anti-inflammatory mechanisms, potentially capturing the host’s biological predisposition to radiation-induced injury (14). An elevated NLR has been associated with adverse outcomes in various cancer types and treatment modalities, including increased risk of radiation-related toxicities (15). This study aimed to develop and validate a comprehensive risk prediction model for RP and radiation-induced pulmonary fibrosis (RIPF) in patients with breast cancer by integrating ipsilateral lung V40 with the pre-radiotherapy NLR, hypothesizing that the combination of physical dose distribution and biological response markers would provide superior predictive performance to either parameter alone, thereby facilitating improved clinical decision-making and individualized treatment strategies.

## Materials and methods

### Study design and patient selection

This retrospective cohort study was conducted at our hospital between May 2018 and August 2020, following approval by the Institutional Review Board. Written informed consent was obtained from all participants for the use of their clinical data in research analyses. The study adhered to the Strengthening the Reporting of Observational Studies in Epidemiology guidelines for observational research. The initial screening identified 198 female patients with histologically confirmed breast cancer who received adjuvant radiotherapy following definitive surgery. The eligibility criteria included the following: (1) pathological diagnosis of invasive breast carcinoma or ductal carcinoma *in situ* (DCIS) requiring postoperative radiotherapy, (2) completion of a prescribed radiotherapy course without interruption exceeding 1 week, (3) availability of complete treatment planning data and dose–volume histograms, (4) baseline pulmonary function within normal limits (predicted forced expiratory volume in 1 second ≥80%, predicted diffusing capacity for carbon monoxide ≥70%), (5) a minimum follow-up duration of 6 months post-radiotherapy with regular imaging surveillance and (6) complete hematological data at specified time points. The exclusion criteria comprised the following: (1) prior thoracic radiotherapy for any indication; (2) pre-existing interstitial lung disease, chronic obstructive pulmonary disease (Global Initiative for Chronic Obstructive Lung Disease stage ≥2) or active asthma requiring daily bronchodilator therapy; (3) bilateral breast cancer or synchronous malignancies; (4) evidence of distant metastases at diagnosis or during treatment; (5) concurrent immunotherapy or targeted therapy during radiotherapy; (6) active autoimmune disorders requiring systemic immunosuppression; (7) pregnancy or lactation during the treatment period; and (8) incomplete dosimetric data or loss to follow-up within 6 months. After applying these criteria, 164 patients were included in the final analysis.

### Treatment protocols

#### Surgical management

Patients underwent either BCS or modified radical mastectomy based on tumor characteristics and patient preference. Sentinel lymph node biopsy was performed for patients who were clinically node negative, with completion axillary lymph node dissection reserved for those with positive sentinel nodes or clinically evident nodal disease. Surgical margins were assessed according to the Society of Surgical Oncology/American Society for Radiation Oncology consensus guidelines, with re-excision performed for positive margins (tumor on ink for invasive carcinoma, <2 mm for DCIS).

#### Systemic therapy

Adjuvant chemotherapy was administered to 140 patients (85.4%) based on the tumor stage, biological subtype and genomic risk assessment when available. Chemotherapy commenced 2–4 weeks postoperatively, with radiotherapy initiated 2–3 weeks after chemotherapy completion. Radiotherapy starts 4–8 weeks after surgery for patients who have not undergone chemotherapy. The chemotherapy regimens included (1) anthracycline-based combinations (doxorubicin/epirubicin plus cyclophosphamide) followed by taxane (AC-T or EC-T) in 68 patients (41.5%), (2) anthracycline plus cyclophosphamide without taxane in 32 patients (19.5%), (3) taxane-based regimens (docetaxel plus cyclophosphamide or carboplatin) in 24 patients (14.6%) and (4) other regimens including single-agent taxane in 16 patients (9.8%). Endocrine therapy was initiated for patients who were hormone receptor positive (n = 132, 80.5%), either concurrently with radiotherapy or immediately following its completion. Hormone epidermal growth factor receptor 2 (HER2)-directed therapy with trastuzumab was administered to 46 patients (28.0%) with HER2-positive disease, typically continuing for a total duration of 1 year.

#### Radiotherapy planning and delivery

All patients underwent computed tomography (CT) simulation in the supine position using individualized immobilization devices. For patients receiving whole breast or chest wall irradiation, a breast board with adjustable arm support was utilized to ensure reproducible positioning. The planning CT was acquired with a 3–5-mm slice thickness from the mandible to the upper abdomen, encompassing all relevant anatomical structures. Intravenous contrast was administered when clinically indicated for improved target delineation.

Target volume delineation followed the Radiation Therapy Oncology Group and European Society for Radiotherapy and Oncology consensus guidelines. The clinical target volume (CTV) for patients undergoing BCS included the entire breast tissue, whereas for patients undergoing mastectomy, it encompassed the chest wall, including surgical scar and drain sites. Regional nodal CTVs were contoured when indicated, including (1) the supraclavicular and infraclavicular nodes (Level III and apical Level II axilla), (2) the internal mammary nodes (IMNs) in the first three intercostal spaces for patients with positive axillary nodes or medial/central tumors and (3) full axilla (Levels I–III) for patients with inadequate axillary surgery or extensive nodal involvement. The planning target volume was generated by adding a 5–7-mm margin to the CTV to account for setup uncertainties and respiratory motion.

Three-dimensional conformal radiotherapy (3D-CRT) was employed for 98 patients (59.8%), utilizing tangential fields for breast/chest wall coverage with appropriate field-in-field modifications to improve dose homogeneity. Intensity-modulated radiotherapy (IMRT) was implemented for 66 patients (40.2%), particularly those requiring comprehensive nodal irradiation or with challenging anatomy precluding acceptable 3D-CRT plans. The prescribed dose was 50 Gy in 25 fractions to the breast/chest wall and regional nodes when indicated. A tumor bed boost of 10–16 Gy was delivered to patients undergoing BCS with high-risk features (age <50 years, positive margins, extensive intraductal component or lymphovascular invasion).

### Dosimetric analysis

Dose–volume histogram parameters were systematically extracted from the treatment planning system for all patients. The ipsilateral lung was defined as the total lung volume minus the CTV to avoid artificial inflation of lung dose metrics. The following dosimetric parameters were analyzed: the MLD and the percentage volumes of ipsilateral lung receiving ≥5 Gy (V5), ≥10 Gy (V10), ≥15 Gy (V15), ≥20 Gy (V20), ≥25 Gy (V25), ≥30 Gy (V30), ≥35 Gy (V35) and ≥40 Gy (V40). Heart dosimetric parameters were also collected for patients with left-sided breast cancer, including the mean heart dose and V25. All dose calculations were performed using heterogeneity corrections with the analytical anisotropic algorithm or Monte Carlo algorithm, depending on the planning system utilized.

### Laboratory assessments

Peripheral blood samples were collected at three standardized time points: (1) within 1 week before surgery (baseline), (2) within 1 week before radiotherapy initiation and (3) 1 month after radiotherapy completion. Complete blood counts with differential were performed using automated hematology analyzers (Sysmex XN-9000, Sysmex Corporation, Japan). The NLR was calculated as the absolute neutrophil count divided by the absolute lymphocyte count. Additional inflammatory markers, including the platelet-to-lymphocyte ratio and systemic immune–inflammation index, were calculated for exploratory analyses. All laboratory assessments were performed in a Clinical Laboratory Improvement Amendments-certified facility with appropriate quality control measures.

### Endpoint assessment

The primary endpoint was the development of RP within 6 months following radiotherapy completion. Secondary endpoints included RIPF (assessed ≥6 months post-radiotherapy) and the severity grading of pulmonary toxicities. Toxicity assessment followed the Common Terminology Criteria for Adverse Events (CTCAE; version 5.0), with grade 1 defined as asymptomatic changes detected only on imaging, grade 2 as symptomatic events limiting instrumental activities of daily living, grade 3 as severe symptoms limiting self-care activities and requiring oxygen, grade 4 as life-threatening respiratory compromise and grade 5 as death. All patients underwent scheduled high-resolution CT at 3 and 6 months, and any new radiographic change, regardless of symptoms, was classified as RP grade 1 according to the CTCAE. Clinical assessment for RP included comprehensive history taking focusing on respiratory symptoms (dyspnea, cough, fever), physical examination and pulse oximetry at each follow-up visit. Chest imaging was performed at 3 and 6 months post-radiotherapy and subsequently at 6-month intervals or when clinically indicated. High-resolution CT was obtained for patients with suspicious symptoms or equivocal chest radiograph findings. All imaging studies were independently reviewed by two radiologists specializing in thoracic imaging, with discrepancies resolved through consensus review with a third radiologist. Pulmonary function tests were performed for patients developing grade ≥2 respiratory symptoms to quantify functional impairment.

### Statistical analysis

Statistical analyses were performed using R software version 4.3.0 (R Foundation for Statistical Computing, Vienna, Austria) and SPSS version 26.0 (IBM Corporation, Armonk, NY, USA). Prior to data collection, a formal sample-size calculation was conducted based on the primary endpoint of developing RP. Assuming an expected RP incidence of 30% in the high-risk group and 10% in the low-risk group, with a two-sided α of 0.05 and power (1-β) of 0.80, a minimum of 142 patients was required. To compensate for a 10% potential loss to follow-up, we aimed to enroll ≥160 patients. The final analytic cohort of 164 patients therefore exceeded the pre-specified requirement and provided adequate power for multivariable modelling. The normality of continuous variables was assessed using the Shapiro–Wilk test and visual inspection of Q–Q plots. Continuous variables were expressed as mean ± standard deviation for normally distributed data or median with interquartile range for skewed distributions. Categorical variables were presented as frequencies and percentages. Between-group comparisons utilized Student’s t-test or the Mann–Whitney U test for continuous variables and chi-squared or Fisher’s exact test for categorical variables, as appropriate. Receiver operating characteristic (ROC) curve analysis was employed to evaluate the discriminatory capacity of individual predictors and determine optimal cutoff values using the Youden index. The AUC with 95% confidence intervals (CIs) was calculated using the DeLong method. Least absolute shrinkage and selection operator (LASSO) regression with 10-fold cross-validation was implemented for variable selection, with the regularization parameter lambda chosen based on the minimum cross-validation error plus one standard error rule to optimize model parsimony. Selected variables were subsequently incorporated into multivariate logistic regression models. Model performance was assessed through multiple metrics including discrimination (AUC), calibration (Hosmer–Lemeshow test and calibration plots) and clinical utility (decision curve analysis). Internal validation utilized 1,000 bootstrap resampling iterations to estimate the optimism-corrected C-index and generate CIs for model parameters. Integrated discrimination improvement (IDI) and net reclassification improvement (NRI) were calculated to quantify the incremental value of adding the NLR to the dosimetric predictors. Interaction terms between V40 and the NLR were tested to explore potential synergistic effects. A nomogram was constructed based on the final multivariate logistic regression model to provide a user-friendly graphical tool for individualized risk prediction in clinical practice. Statistical significance was defined as P < 0.05 for all analyses.

## Results

### Patient characteristics

The study cohort comprised 164 female patients with breast cancer with a median age of 50 years (range: 22–75 years). Left-sided breast cancer was present in 87 patients (53.0%), and 77 (47.0%) had right-sided disease. The predominant histological subtype was invasive ductal carcinoma (90.2%), followed by invasive lobular carcinoma (6.7%). Most patients presented with stage I disease (63.4%), with 25.6% having stage II and 6.7% stage III disease. Hormone receptor-positive disease was observed in 80.5% of patients, with luminal A representing the most common molecular subtype (60.4%). Regional lymph node irradiation was administered to 96 patients (58.5%), and 69 patients (42.1%) received IMN irradiation based on risk stratification (**Table 1**).

**Table 1:** Baseline clinical and pathological characteristics (N=164).

Characteristic	Value
Age, years	
Median (range)	50 (22-75)
≥50 years, n (%)	85 (51.8%)
Clinical stage, n (%)	
I	104 (63.4%)
II	42 (25.6%)
III	11 (6.7%)
Molecular subtype, n (%)	
Luminal A	99 (60.4%)
Luminal B	26 (15.9%)
HER2-enriched	21 (12.8%)
Triple-negative	18 (11.0)
Estrogen receptor positive, n (%)	122 (74.4%)
Progesterone receptor positive, n (%)	111 (67.7%)
HER2 positive, n (%)	46 (28.0)
Ki-67 ≥10%, n (%)	115 (70.1%)
Breast-conserving surgery	83 (50.6%)
Mastectomy	81 (49.4%)
Regional nodal irradiation, n (%)	96 (58.5%)
Internal mammary node irradiation, n (%)	69 (42.1%)

### Incidence and characteristics of radiation-induced lung toxicity

**Figure 1:**
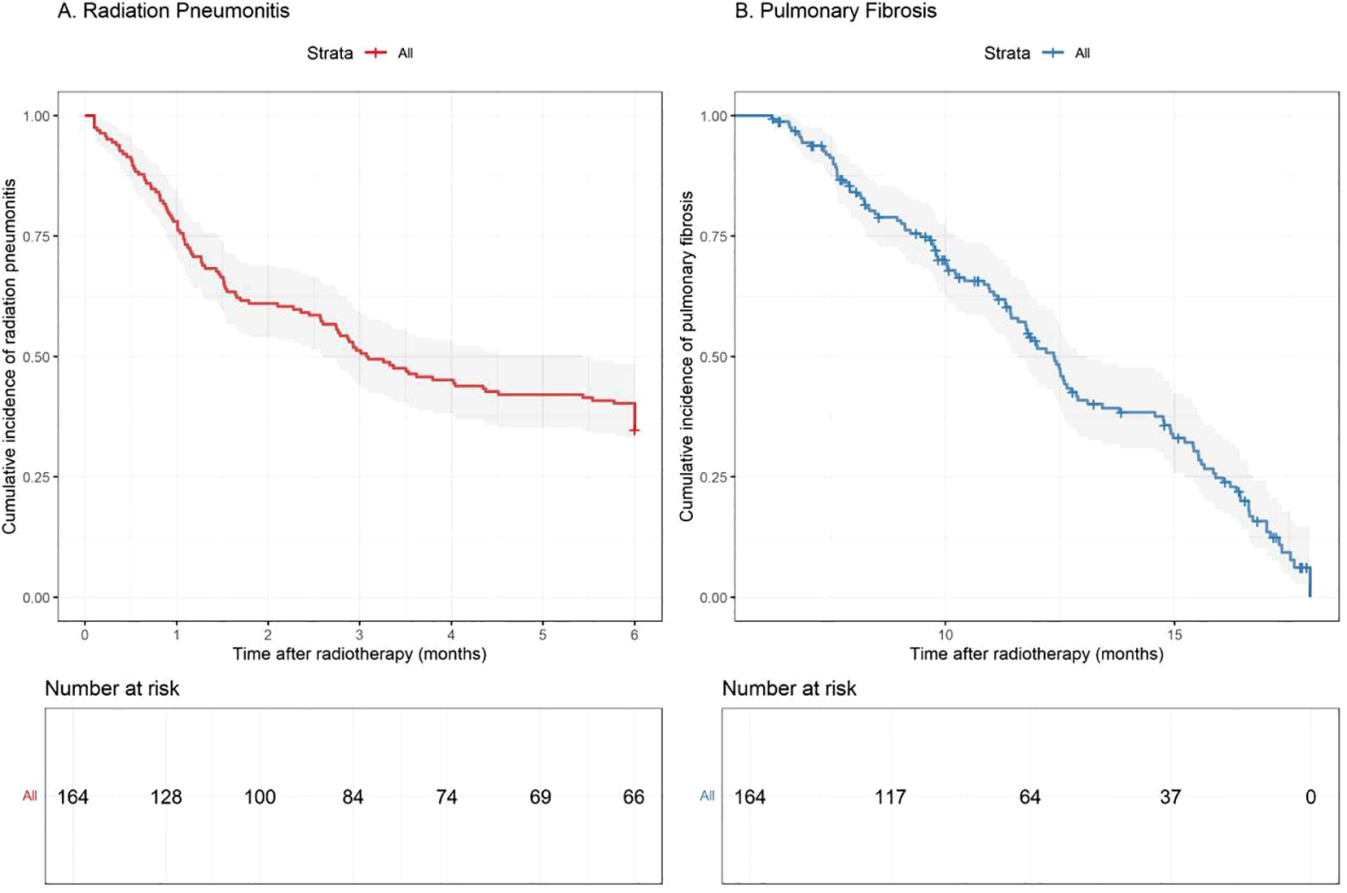
Kaplan-Meier curves showing the cumulative incidence of **(A)** radiation pneumonitis and **(B)** pulmonary fibrosis over time following radiotherapy completion.

Among the 164 patients analyzed, 107 (65.2%) developed RP of any grade within 6 months post-radiotherapy. The severity distribution included 103 patients (62.8%) with grade 1 RP and 4 patients (2.4%) with grade 2 RP. No cases of grade 3 or higher RP were observed during the study period. The median time to RP onset was 2.3 months (range: 0.8–5.7 months) after radiotherapy completion. Anatomically, the left upper lobe was most frequently affected (45 cases), followed by the right upper lobe (38 cases), correlating with the radiation field distribution. Radiation-induced pulmonary fibrosis was documented in 118 patients (72.0%) at ≥6-months follow-up, all classified as grade 1 with characteristic imaging findings but without functional impairment. The median follow-up duration was 18 months (range: 6–48 months), allowing adequate time for late toxicity assessment (**Figure 1**).

### Dosimetric parameters analysis

The mean ipsilateral lung volume was 1,156.3 ± 287.5 cm³, with substantial individual variation reflecting anatomical differences. The MLD averaged 12.8 ± 3.2 Gy across the cohort (**Table 2**). Analysis of dose–volume parameters revealed a progressive decrease in lung volume percentages receiving higher doses, with V5 averaging 48.6% and V40 averaging 9.89%. Notably, patients receiving IMN irradiation demonstrated significantly higher V20 (24.3% vs 20.6%, P = 0.008) and V40 (11.8% vs 9.2%, P = 0.003) than those without IMN treatment.

**Table 2:** Dosimetric parameters of ipsilateral lung.

Parameter	Mean ± SD	Median (IQR)	Range
Lung volume (cm³)	1156.3 ± 287.5	1142.8 (965.2-1324.5)	687.4-1876.3
Mean lung dose (Gy)	12.8 ± 3.2	12.4 (10.6-14.8)	6.8-21.3
V5 (%)	48.6 ± 12.3	47.2 (39.8-56.7)	24.3-76.8
V10 (%)	35.4 ± 9.8	34.6 (28.3-41.2)	15.6-58.9
V15 (%)	27.8 ± 8.2	27.1 (21.9-33.4)	11.2-46.7
V20 (%)	21.5 ± 6.8	20.8 (16.4-26.2)	7.8-37.4
V25 (%)	16.3 ± 5.9	15.7 (12.1-19.8)	4.3-29.6
V30 (%)	12.7 ± 5.1	12.2 (8.9-15.8)	2.1-24.3
V35 (%)	10.1 ± 4.4	9.6 (6.8-12.9)	0.8-19.7
V40 (%)	9.89 ± 3.76	9.45 (7.12-11.98)	0.3-17.8

### Predictive performance of dosimetric parameters

**Figure 2:**
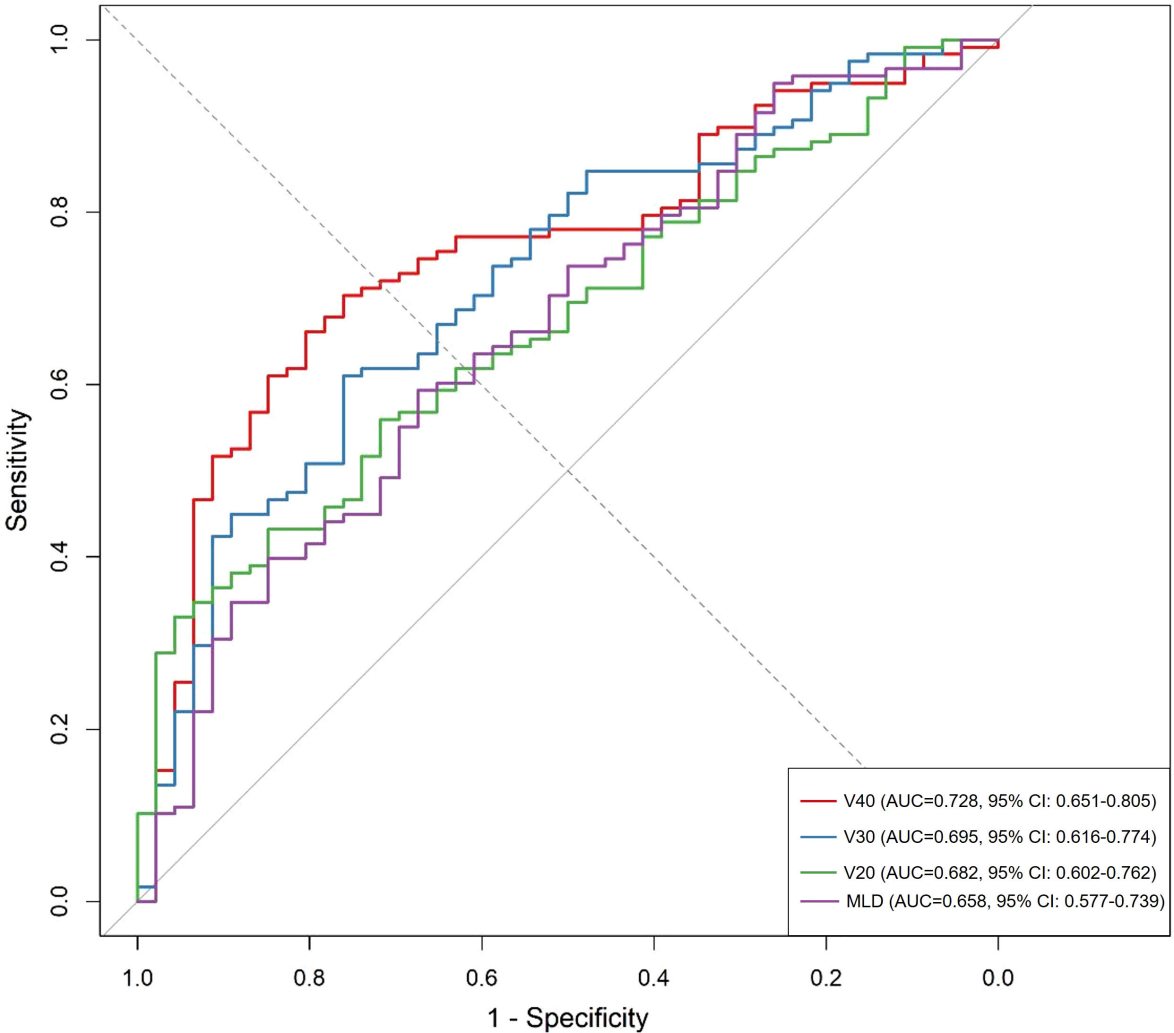
Receiver operating characteristic curves comparing the predictive performance of dosimetric parameters (V40, V30, V20, and MLD) for radiation-induced pulmonary fibrosis.

The ROC curve analysis evaluated the discriminatory capacity of various dosimetric parameters for predicting RIPF. Among these parameters, V40 demonstrated superior predictive performance with an AUC of 0.728 (95% CI: 0.651–0.805), followed by V30 (AUC = 0.695, 95% CI: 0.616–0.774) and V20 (AUC = 0.682, 95% CI: 0.602–0.762). The optimal cutoff value for V40 was determined to be 10.45%, yielding a sensitivity of 68.6% and specificity of 73.9%. The MLD showed modest predictive value (AUC = 0.658, 95% CI: 0.577–0.739) (**Figure 2**).

### Dynamic changes and predictive value of the neutrophil-to-lymphocyte ratio

The NLR demonstrated a progressive increase from baseline to post-radiotherapy assessment, reflecting a cumulative inflammatory burden from sequential treatments. Patients who developed RP exhibited significantly higher NLR values at all time points than those without RP. The pre-radiotherapy NLR showed the strongest association with RP development (4.28 ± 3.12 vs 2.45 ± 1.56, P < 0.001). The ROC analysis revealed that the pre-radiotherapy NLR possessed significant predictive value for RP (AUC = 0.685, 95% CI: 0.605–0.765), with an optimal cutoff of 2.82 providing 62.6% sensitivity and 71.9% specificity. By contrast, the pre-surgical NLR (AUC = 0.598) and post-radiotherapy NLR (AUC = 0.572) demonstrated inferior predictive performance (**Table 3**).

**Table 3:** Temporal evolution of neutrophil-to-lymphocyte ratio.

Time point	Overall (N=164)	RP group (n=107)	Non-RP group (n=57)	P-value
Pre-surgery	3.21 ± 1.85	3.48 ± 2.01	2.71 ± 1.42	0.012
Pre-radiotherapy	3.64 ± 2.78	4.28 ± 3.12	2.45 ± 1.56	<0.001
Post-radiotherapy	4.12 ± 2.93	4.56 ± 3.24	3.29 ± 2.18	0.008

### Multivariate analysis and model development

The LASSO regression with 10-fold cross-validation was applied to 40 candidate variables, identifying six features with non-zero coefficients: V40, pre-radiotherapy NLR, regional nodal irradiation, age, Ki-67 index and MLD. These selected variables were evaluated in multivariate logistic regression analysis.

The final prediction model incorporated four independent risk factors: V40 ≥10.45%, pre-radiotherapy NLR ≥2.82, regional nodal irradiation and age ≥50 years. The logistic regression equation was formulated as follows:


Logit(P)=−3.256+1.176×V40+0.940×NLR+0.756×Nodal_RT+0.598×Age

(**Table 4**).

**Table 4:** Multivariate logistic regression analysis for radiation pneumonitis.

Variable	Odds Ratio	95% CI	P-value
V40 ≥10.45%	3.24	1.78-5.89	<0.001
Pre-RT NLR ≥2.82	2.56	1.42-4.61	0.002
Regional nodal irradiation	2.13	1.18-3.84	0.012
Age ≥50 years	1.82	1.05-3.15	0.033
Ki-67 ≥10%	1.54	0.87-2.72	0.138
Mean lung dose (per Gy)	1.08	0.98-1.19	0.124

#### Model performance evaluation

The combined prediction model demonstrated superior discriminatory capacity with an AUC of 0.816 (95% CI: 0.748–0.884), significantly outperforming individual predictors including V40 alone (AUC = 0.728, P = 0.012) and NLR alone (AUC = 0.685, P = 0.018). Bootstrap internal validation with 1,000 iterations yielded an optimism-corrected C-index of 0.803, indicating robust model performance.

**Figure 3:**
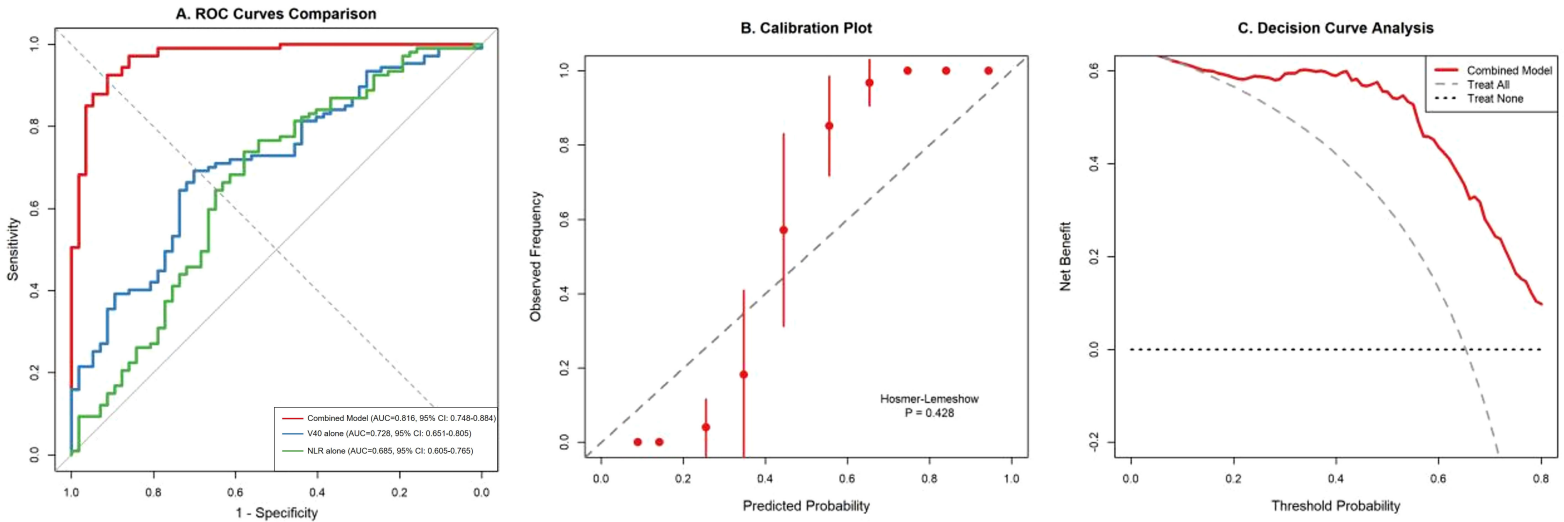
**(A)** ROC curves comparing the combined model with individual predictors. **(B)** Calibration plot demonstrating agreement between predicted and observed RP probabilities. **(C)** Decision curve analysis showing net benefit across threshold probabilities.

Calibration assessment revealed excellent agreement between predicted and observed probabilities (Hosmer–Lemeshow test P = 0.428). Decision curve analysis demonstrated that the combined model provided greater net benefit than the treat-all or treat-none strategies across threshold probabilities ranging from 10% to 80% (**Figure 3**).

#### Model improvement metrics

The addition of the NLR to the base dosimetric model resulted in significant improvements in predictive performance: IDI was 0.082 (P = 0.003), and NRI was 0.354 (P < 0.001). Further incorporation of clinical factors increased IDI to 0.131 (P < 0.001).

#### Interaction effects

**Figure 4:**
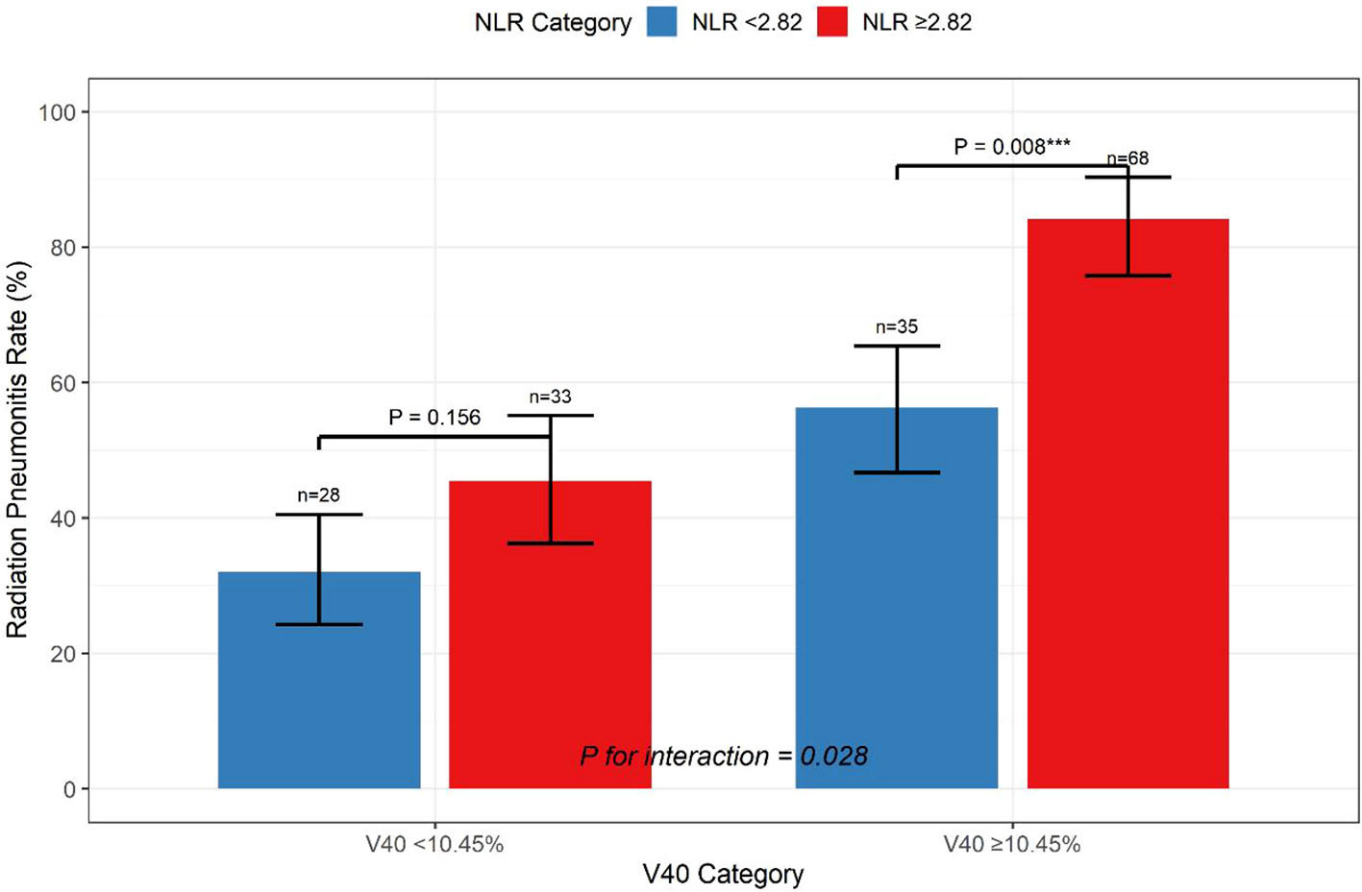
Stratified analysis showing the interaction between V40 and NLR in predicting radiation pneumonitis risk. *** indicates significant difference.

Significant interaction was observed between V40 and the NLR (P for interaction = 0.028). Among patients with V40 ≥ 10.45%, those with a high NLR (≥2.82) had an RP incidence of 84.2% compared with 56.3% in the low NLR group (P = 0.008). Conversely, in patients with V40 < 10.45%, the impact of the NLR was attenuated (45.5% vs 32.1%, P = 0.156) (**Figure 4**).

#### Nomogram development

**Figure 5:**
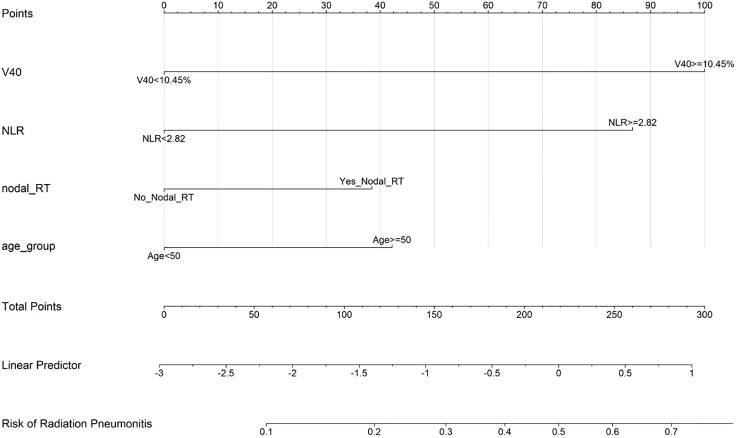
Clinical nomogram for predicting radiation pneumonitis risk in breast cancer patients. Points are assigned for each variable, with the total points corresponding to the predicted probability of developing radiation pneumonitis.

Based on the multivariate logistic regression model, a clinical nomogram was constructed to facilitate individualized risk assessment. The nomogram integrates the four significant predictors (V40, pre-radiotherapy NLR, regional nodal irradiation and age) to generate personalized RP risk estimates ranging from <10% to >80% (**Figure 5**).

## Discussion

This study successfully developed and validated a comprehensive risk prediction model for RP in patients with breast cancer by integrating dosimetric parameters with inflammatory biomarkers. The combined model incorporating ipsilateral lung V40 and the pre-radiotherapy NLR demonstrated superior predictive performance (AUC = 0.816) to traditional single-parameter approaches, providing a practical tool for personalized risk stratification in clinical practice (16).

Our finding that V40 outperforms V20 and the MLD in predicting RP is consistent with reports on partial lung irradiation for breast radiotherapy, where high-dose volumes correlate more strongly with cior breast radiotherapy studlinical pneumonitis than the mean-dose or low-dose bath metrics (17). This observation aligns with recent evidence suggesting that high-dose–volume parameters may better reflect the risk of RP in partial lung irradiation scenarios. The V40 cutoff of 10.45% identified in our study is consistent with that of 8%–12% reported in the contemporary literature, supporting its validity as a clinically relevant threshold (18). To our knowledge, no prior breast radiotherapy study has validated a composite model integrating V40 with the NLR; our results suggest that combining dosimetric and inflammatory domains yields clinically meaningful gains in discrimination and net benefit.

The enhanced predictive capacity of V40 likely reflects the unique dose distribution patterns in breast cancer radiotherapy, in which the anterior–superior portion of the ipsilateral lung receives substantially higher doses than whole-lung irradiation scenarios (19). Modern treatment planning studies have demonstrated that although techniques such as IMRT and volumetric modulated arc therapy can reduce high-dose volumes (V30, V40), they may increase low-dose bath effects (V5, V10), underscoring the importance of monitoring high-dose parameters in treatment optimization (20).

The clinical implications of our V40 findings extend beyond risk prediction to inform treatment planning strategies. For patients requiring comprehensive nodal irradiation, particularly including the internal mammary chain, achieving V40 constraints below 10.45% may necessitate advanced techniques, such as deep inspiration breath-hold (DIBH), proton therapy or modified field arrangements (21). Our subgroup analysis revealed that patients receiving IMN irradiation had significantly higher V40 values (11.8% vs 9.2%), emphasizing the need for careful consideration of target volume selection and planning techniques in high-risk patients.

The identification of the pre-radiotherapy NLR as an independent predictor of RP provides important insights into the role of systemic inflammation in radiation-induced lung injury (22). The NLR cutoff of 2.82 established in our cohort closely approximates values reported in lung cancer studies, suggesting consistent inflammatory thresholds across thoracic malignancies. The cutoff values identified in this study (NLR ≥ 2.82 and V40 ≥ 10.45%) were generated through data-driven ROC optimization within our single-institution cohort. Because these thresholds may shift in populations with different baseline characteristics, treatment techniques or ethnic distributions, they should be regarded as tentative guides rather than universal constants. External validation and, if necessary, recalibration are required before the proposed cutoffs are adopted in other clinical settings. The progressive increase in the NLR from baseline to post-treatment assessment reflects the cumulative inflammatory burden of multimodal therapy, with pre-radiotherapy values capturing the optimal predictive window (23).

The mechanistic basis for the NLR’s predictive value likely involves multiple pathways: neutrophils release reactive oxygen species and proteolytic enzymes that amplify radiation-induced tissue damage, whereas lymphocyte depletion compromises tissue repair mechanisms and immunoregulatory functions (24). Our observation of significant NLR–V40 interaction suggests that inflammatory status may modulate the dose–response relationship, with high baseline inflammation potentially lowering the threshold for radiation injury.

The temporal dynamics of the NLR observed in our study, increasing from surgery to chemotherapy and radiotherapy, highlight the importance of timing in biomarker assessment (25). The predictive value of the pre-radiotherapy NLR compared with baseline or post-treatment values suggests that the inflammatory milieu immediately preceding radiation exposure critically influences toxicity risk. This finding has practical implications for treatment sequencing and the potential use of anti-inflammatory interventions in high-risk patients.

The significant interaction between V40 and NLR represents a key finding with important clinical implications. Among patients with high V40 (≥10.45%), the presence of an elevated NLR nearly doubled the RP risk (84.2% vs 56.3%), whereas the NLR had minimal impact in the low V40 group (26). This interaction suggests that dosimetric and inflammatory factors operate synergistically rather than independently, supporting the biological rationale for multimodal risk assessment. A critical consideration in interpreting our findings is the potential confounding effect of perioperative and chemotherapy-related inflammatory responses on NLR values. Patients with breast cancer experience a well-documented, time-dependent inflammatory cascade: surgical trauma induces an acute-phase response that typically peaks within 48–72 hours and may persist for 7–10 days, whereas anthracycline- and taxane-based chemotherapy regimens produce a secondary, more prolonged neutrophilic response that can last 5–7 days after each cycle. These transient spikes can artifactually elevate the NLR independent of any underlying host immune phenotype. Nevertheless, we acknowledge that subclinical or residual inflammatory activity could still bias measurements. Future prospective studies should incorporate serial NLR sampling across the peri-treatment timeline and apply trajectory-based modelling to better isolate the host-specific inflammatory set point from transient therapy-induced fluctuations.

Recent advances in machine learning approaches for RP prediction have emphasized the value of integrating multiple data domains, including clinical, dosimetric and biological parameters (27). The LASSO-based variable selection approach in this study effectively identified the most informative predictors while avoiding overfitting, demonstrating the utility of regularized regression techniques in developing parsimonious yet accurate prediction models.

The substantial improvement in model performance metrics, including an IDI of 0.131 and NRI of 0.354, quantifies the added value of incorporating inflammatory markers into traditional dosimetric models (28). These improvements translate to meaningful clinical benefits, as demonstrated by decision curve analysis showing increased net benefit across a wide range of threshold probabilities relevant to clinical decision-making.

Our nomogram offers a practical, clinically applicable tool for individualized risk assessment (29). Based on the predicted risk scores, patients can be stratified into low (<30%), intermediate (30%–60%) and high (>60%) risk categories, enabling tailored prevention and monitoring strategies.

For high-risk patients, management strategies may include (1) planning modifications such as DIBH or altered fractionation, (2) prophylactic agents (pentoxifylline, vitamin E, inhaled corticosteroids), (3) intensified surveillance during the 2–4-month high-risk window post-radiotherapy and (4) patient education on early symptom recognition (30).

Integrating our model into clinical workflows may support shared decision-making, especially in patients with borderline indications for nodal irradiation, where the risk–benefit balance is critical. Furthermore, the model could guide clinical trial design by enabling risk-stratified randomization and enrichment strategies for studies evaluating pneumonitis prevention interventions. Fourth, although the median follow-up of 18 months is sufficient to capture most cases of early RP and to document initial fibrotic changes, longer observation (>2 years) would further strengthen the assessment of late pulmonary toxicity and the durability of our model’s predictive value. Finally, this retrospective, single-center design is subject to selection and information bias; treatment indications, imaging frequency and grading criteria may influence event rates. Residual confounding from systemic therapies, smoking, comorbid pulmonary disease and baseline function cannot be fully excluded. Thresholds for V40 and the NLR are data derived and require external validation to ensure transportability across techniques and population. Prospective, multicenter validation with standardized toxicity adjudication and the incorporation of pulmonary function and patient-reported outcomes is warranted to refine thresholds and confirm clinical utility.

## Conclusion

This study successfully developed and validated a prediction model for RP in patients with breast cancer by integrating ipsilateral lung V40 with the pre-radiotherapy NLR. The combined model demonstrated superior predictive performance to single-parameter approaches, with excellent discrimination (AUC = 0.816) and calibration. The identification of substantial interaction between dosimetric and inflammatory predictors highlights the multifactorial nature of radiation-induced lung injury. Clinical implementation of this model through the provided nomogram enables personalized risk stratification, facilitating optimized treatment planning and preventive strategies that may ultimately reduce RP-related toxicity and preserve patients’ quality of life. Future prospective validation studies and interventional trials are warranted to confirm the model’s utility in improving patient outcomes through precision medicine approaches in breast cancer radiotherapy.

## Data Availability

The original contributions presented in the study are included in the article/supplementary material. Further inquiries can be directed to the corresponding authors.
